# Dynamic adjustment algorithm of equipment maintenance cycle based on substation state operation and maintenance

**DOI:** 10.1371/journal.pone.0349132

**Published:** 2026-05-22

**Authors:** Yuping Yan, Fangfang Zhou, Zhongyue Cai, Hongzhi Lu

**Affiliations:** Guangdong Power Grid Co., Ltd., Guangzhou, China; Aalto University, FINLAND

## Abstract

To improve the timeliness and effectiveness of equipment maintenance and ensure the stable operation of power systems, this paper proposes a dynamic adjustment algorithm for equipment maintenance cycles based on substation condition-based operation and maintenance. The algorithm builds the operation and maintenance management framework of smart substation, adopts the distributed multi-sensor mode to sense the operation and maintenance status data of substation equipment, Based on the operation and maintenance status data of substation equipment, analyze the planned maintenance cost and failure cost of substation equipment, determine the dynamic adjustment objective function of the equipment maintenance cycle based on these two costs, and set the constraint condition of the dynamic adjustment target function of the equipment maintenance cycle from the perspective of the total maintenance plan, the coupling constraint between the maintenance start time and the daily state variables of the maintenance plan, after solving the dynamic adjustment objective function of the equipment maintenance cycle, to obtain the optimal dynamic adjustment parameters of the equipment maintenance cycle, ensure that the planned maintenance cost and failure cost of the adjustment are the lowest. Experimental results show that the proposed algorithm effectively perceives substation equipment condition data, dynamically adjusts maintenance cycles, shortens maintenance durations, and improves the reliability coefficient of substation equipment. The application effect is relatively significant.

## 1 Introduction

In power system, the state operation and maintenance of substation is the key link to ensure the stable operation of the whole system. With the continuous upgrading of power equipment and the expansion of power grid scale, the traditional substation state operation and maintenance mode has been unable to meet the needs of modern power grid [1]. In order to deal with this challenge, the dynamic adjustment algorithm of equipment maintenance cycle based on substation condition operation and maintenance came into being. This algorithm can dynamically adjust the maintenance cycles of substation equipment based on their real-time operating status and historical data, thereby achieving more efficient and intelligent operation and maintenance management for substations [2]. At present, the significance of the dynamic adjustment algorithm of equipment maintenance cycle is not only to improve the operating efficiency and reliability of equipment, but also to adapt to future development needs [3]. With the continuous upgrading and transformation of power system, dynamic regulation algorithm can meet the needs of future development and improve the intelligent level of power system. At the same time, the algorithm can be flexibly adjusted according to the actual situation, and has strong practicability and scalability. At home and abroad, some achievements have been made in the research on the dynamic adjustment algorithm of substation condition operation and maintenance and equipment maintenance cycle. Experts and scholars at home and abroad have proposed a variety of dynamic adjustment algorithms for equipment maintenance cycle, such as Compare et al. [4] proposed a state-based equipment maintenance life cycle adjustment method. This method monitors the operation status of the equipment in real time, obtains the key performance parameters and fault modes, combines the historical maintenance data of the equipment and expert knowledge, evaluates the health status of the equipment, and predicts the remaining life and potential failure risk. According to the different status of the equipment, the existing maintenance cycle is dynamically adjusted. Betti et al. [5] proposed a method for condition monitoring and maintenance cycle adjustment of hydropower station equipment. This method uses sensors and other equipment to collect real-time operation data of substation equipment, optimize and adjust the maintenance cycle according to the current equipment status of the substation, reduce the maintenance cycle time and improve the maintenance frequency. M’Halla [6] proposed the static adjustment method of equipment maintenance scheduling. After this method is divided according to the emergency degree of equipment use, the equipment maintenance cycle is dynamically adjusted by dispatching different maintenance periods. After adjustment, the adjusted equipment maintenance cycle is longer and the frequency of equipment maintenance is more. Coit et al. [7] designed an equipment dynamic maintenance cycle adjustment model based on deep reinforcement learning. This method can obtain the results of equipment dynamic maintenance cycle adjustment by establishing a mathematical model of substation equipment operation status, establishing a maintenance cycle adjustment function covering the performance standards of substation equipment, and introducing and introducing a gradient algorithm of deep deterministic strategy to solve it. Bagnato and Krasnodebska [8] proposed an optimization method for equipment maintenance life cycle based on cloud computing, which dynamically adjusts the equipment maintenance life cycle in an optimized way.

In response to the aforementioned challenges, this paper proposes a dynamic adjustment algorithm for equipment maintenance cycles in smart substations. Compared with existing research, the core innovations of this work are reflected in the following three aspects:

(1) A Multi-Dimensional Priority Comprehensive Evaluation Mechanism.

While existing Condition-Based Maintenance (CBM) can adjust cycles based on equipment status, it lacks the capability to handle complex constraints among multiple devices. Reliability-Centered Maintenance (RCM), though considering equipment criticality, struggles to achieve real-time dynamic adjustments [9]. This paper integrates five types of priority rules—equipment criticality, health status, window urgency, maintenance duration, and mutual exclusion or collaboration relationships—into the objective function using a weighted multi-attribute decision-making method. This approach achieves a transition from “single-device state response” to “multi-device collaborative optimization.”

(2) Dynamic Coupling of State Perception and Maintenance Models.

This paper embeds real-time monitored equipment state parameters directly into the equipment failure rate evolution model characterized by the age reduction factor. This establishes a dynamic feedback loop between state data and maintenance decisions, enabling maintenance cycle adjustments to respond in real time to changes in equipment health status. This drives the decision-making upgrade from “scheduled maintenance” to “precise condition-based maintenance.”

(3) A Two-Stage Solving Strategy Combining Genetic Algorithms and Nonlinear Programming.

Addressing the high-dimensional, nonlinear, and multi-constrained nature of the model, this paper proposes a collaborative solving mechanism that combines global search via genetic algorithms with local optimization via nonlinear programming. This approach balances global exploration capabilities with local optimization accuracy, significantly improving computational efficiency while ensuring solution quality [10].

In summary, the proposed algorithm achieves organic integration of multi-objective and multi-constraint modeling at the conceptual level, overcomes the efficiency bottlenecks of traditional methods at the solving level, and realizes fundamental innovation in the dynamic coupling of state perception and decision optimization. In contrast to traditional CBM, which typically optimizes maintenance cycles for individual equipment without considering multi-device coupling constraints, the proposed framework explicitly embeds mutual exclusion, synergy, window, and resource constraints into the objective function. Unlike RCM, which relies on static risk tables and fixed intervals, this method dynamically couples real-time condition data with an age-reduction-factor failure model, enabling real-time cycle adjustments. Furthermore, the two-stage genetic algorithm-nonlinear programming solver addresses the high-dimensional, nonlinear optimization problem that neither CBM nor RCM can effectively handle. Thus, the proposed approach achieves a transition from single-device state response and static reliability rules to multi-device collaborative dynamic optimization.

The paper constructs an integrated maintenance cycle dynamic adjustment framework for intelligent substations, breaking through the limitations of traditional state based maintenance (CBM) that only relies on single device state information. It embeds five priority rules, including device criticality, health status, window urgency, maintenance duration, and mutual exclusion/collaboration relationships, into the objective function through a weighted multi-attribute decision-making method; By coupling the real-time monitored state parameters directly into the equipment failure rate evolution model through an age decreasing factor, a dynamic feedback loop between state data and maintenance decisions is established; A collaborative solution strategy combining genetic algorithm global search and nonlinear programming local refinement was designed to address the high-dimensional, nonlinear, and multi constraint characteristics of the model, significantly improving computational efficiency while ensuring solution quality. Compared with existing research, this framework does not rely on black box models and large-scale annotated data, nor is it limited to static dependency relationships or single system testing strategies, providing an interpretable, efficient, and robust engineering solution for substation state operation and maintenance.

## 2 Algorithm for dynamic adjustment of equipment maintenance cycle

### 2.1 Intelligent substation operation and maintenance management framework construction

**Fig 1:**
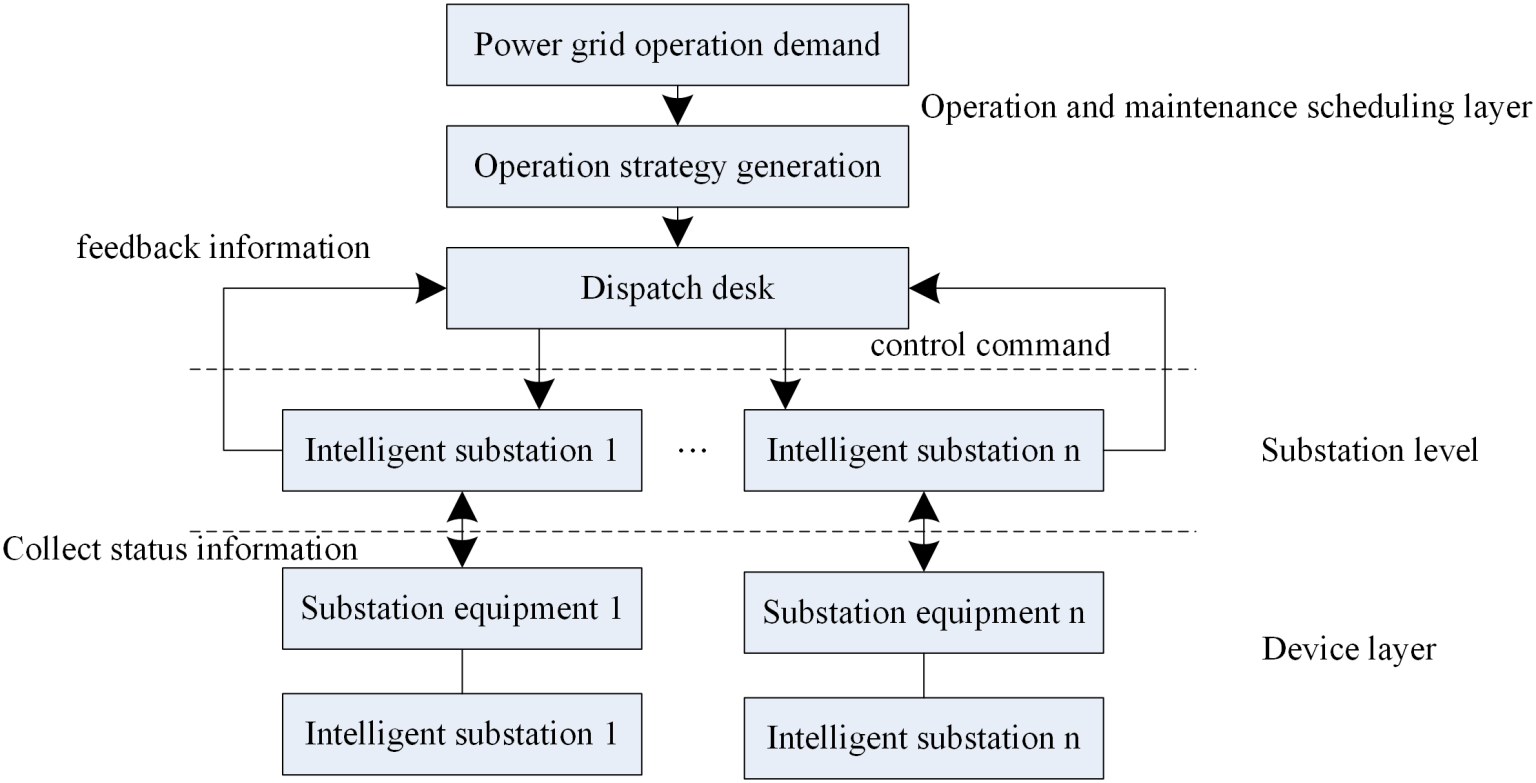
Intelligent substation operation and maintenance management framework.

Substation in the power system operation and management plays an important role, in recent years, the development of intelligent substation is more by many scientific research team as well as the relevant departments of attention. Intelligent substation operation and management is intelligent, friendly and convenient, intelligent substation monitoring, scheduling by the scheduling department is responsible for the management of the intelligent substation and its intelligent equipment by the substation operation and maintenance personnel jurisdiction [11]. Intelligent equipment detection sends signals to the dispatching station, the controller analyzes the signals found, combines the operation mode and relay protection strategy, and sends scheduling instructions to the intelligent substation maintenance personnel for operation. Intelligent substation consists of intelligent electrical equipment, and each intelligent substation has an intelligent processing core. The processing core can receive scheduling commands and decompose them into single-step operation steps. Before operation, the processing core can also feedback the information of the intelligent equipment to the scheduling center and prompt the possible risks caused by the operation, and after getting confirmation from the scheduling, the operation will be executed directly, and the results will be returned to the scheduling after the execution of the commands. Each instruction, operation step and operation result will be automatically recorded in the database of the processing nucleus. The design of intelligent substation operation and maintenance management framework is shown in Fig 1.

### 2.2 Substation equipment operation and maintenance state data sensing methods

**Fig 2:**
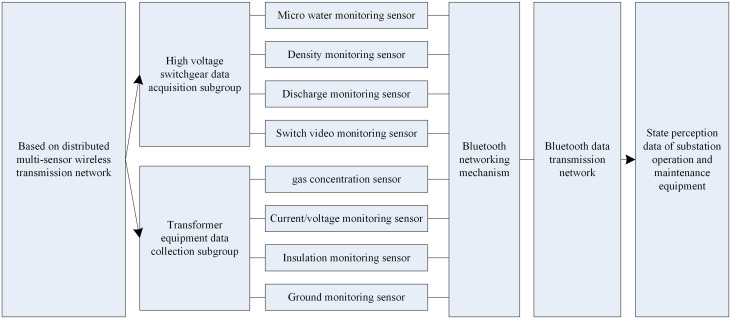
Equipment state perception process for substation state operation and maintenance.

Utilizing distributed multi-sensors to obtain substation operation and maintenance status data sensing, based on substation equipment details, substation operation and maintenance status data sensing process is shown in Fig 2.

The equipment state sensing of substation state operation and maintenance is based on distributed multi-sensor wireless sensing network. For substation high-voltage switchgear and transformer equipment, distributed multi-sensors such as micro-water monitoring sensors, density monitoring sensors, discharge monitoring sensors, switching video monitoring sensors, gas concentration sensors, voltage/current sensors, etc., are used to obtain the equipment state data of substation high-voltage switchgear and transformer equipment, and a Bluetooth data transmission network is established based on Bluetooth networking mechanism. Equipment status data, in accordance with the Bluetooth networking mechanism to establish a Bluetooth data transmission network, the use of the network will be sent to the substation status operation and maintenance data to the intelligent substation operation and maintenance management framework, substation operation and maintenance status data [12], for the dynamic adjustment of the equipment maintenance cycle of the basic data.

### 2.3 Cost analysis of equipment overhaul cycles

MakeG represent the substation operation and maintenance status data set, based on which the cost of substation operation and equipment maintenance cycle is analyzed.

(1) Cost of planned maintenance

The planned maintenance cost is the sum of the direct cost of maintenance and the loss cost caused by production stagnation [13]. Based on the historical data of the enterprise, the single maintenance and overhaul cost $\left(U_{mL},U_{mH}\right)$ corresponding to each maintenance level can be obtained, where $U_{mL}$ represents the low level (such as L level) single maintenance and overhaul cost, and $U_{mH}$ represents the high level (such as H level) single maintenance and overhaul cost. Assuming that the operating time interval of the device is[0,T], assume in the zone[0,T]there is totally n overhaul, of which L level x times, H level y times (Satisfy x+y=n). The L and H levels here can be classified based on factors such as the complexity and importance of the equipment. For example, for transformers, routine appearance inspections, simple parameter testing, etc. can be classified as L level, while complex operations involving internal winding detection, key component replacement, etc. can be classified as H. Then in the interval [0,T], the cost $U_{m}$ of planned overhauls is:


$$\begin{array}{c} U_{m}=\left[\sum_{i=1}^{x}U_{mLi}+\sum_{i=1}^{y}U_{mHi}\right]\cdot G \end{array}$$ (1)

Among them, $U_{mLi}=U_{mL}$ (the cost of each L-level maintenance is the same), $U_{mHi}=U_{mH}$ (the cost of each H-level maintenance is the same), and G is a coefficient that considers the impact of operation and maintenance status data (for example, when the overall operation and maintenance status of the equipment is good, G tends to be close to 1, and if there are potential risk factors, G can be appropriately amplified to reflect the uncertainty of costs).

(2) Cost of failure

The cost of failure is the sum of the direct cost of failure repairs and the cost of losses due to production stoppage. If the failure rate of the equipment is λ(t) (λ(t) is a function of time t, which can be fitted by statistical methods using historical fault data of the equipment, such as exponential distribution $\lambda \left(t\right)=\lambda_{\text{0}}e^{-\lambda_{\text{0}}t}$, where $\lambda_{\text{0}}$ is the constant of the fault rate, which can be estimated based on the historical fault interval time of similar equipment), the cost of a single failure is $U_{r}$, within the i th maintenance cycle, the cost of failure $\left[t_{i-1},t_{i}\right]$, $U_{ri}=\int_{t_{i-1}}^{t_{i}}\lambda_{i}\left(t\right)U_{r}dt$. The maintenance cycle division here can be based on the operating characteristics of the equipment, such as dividing it according to the cumulative operating hours of the equipment, with every 1000 hours being one cycle. Then in [0,T], the cost $\sum_{I=1}^{n}U_{Ri}$ of maintenance failures is:


$$\begin{array}{c} \sum_{I=1}^{n}U_{Ri}=Ur\left[\int_{\text{0}}^{t_{i}}\lambda_{\text{1}}\left(t\right)dt+\int_{t_{\text{1}}}^{t_{\text{2}}}\lambda_{\text{1}}\left(t\right)dt+\cdots +\int_{t_{n-1}}^{t_{n}}\lambda_{n}\left(t\right)dt\right]\cdot G \end{array}$$ (2)

Where $\lambda_{i}\left(t\right)$ represents the failure rate function within the i-th maintenance cycle (assuming a stable failure rate within each cycle, $\lambda_{i}\left(t\right)=\lambda$).

The parameter values in the above cost model have a significant impact on the optimization results, and their determination is based on the following justifications:

The costs for single maintenance and inspection, $U_{mL}$ and $U_{mH}$, are derived from a statistical analysis of historical maintenance data at the substation over the past five years. The average cost for L-level maintenance (routine inspection) is 0.8 million yuan, while the average cost for H-level maintenance (component replacement) is 2.5 million yuan. The average value of the maintenance bills for this substation over the past five years.

The cost coefficient G ranges within [0.8, 1.2] and is used to reflect the influence of equipment operational status on actual maintenance costs.

The single failure cost $\sum_{I=1}^{n}U_{Ri}$ includes direct repair costs and outage losses. Based on historical fault records and the load characteristics of the substation’s power supply area, this value is set at 3.5 million yuan.

These parameter values are generally consistent with reference values from similar studies. Moreover, their stability will be further validated through sensitivity analysis (see Section 3.7).

The cost of a single maintenance is derived from the statistical average of the historical maintenance bills of the substation over the past 5 years, covering direct expenses such as labor, spare parts, and testing. The cost of a single failure is calculated based on the maintenance records of the past 3 years and the power outage losses, where the power outage losses are calculated based on the average load shortage cost of the power supply area of the substation. The range of cost coefficient values [0.8, 1.2] is calibrated through expert interviews and maintenance records: when the equipment is in good condition, the cost coefficient is taken as 1.0, and when there is potential risk, it is taken as 1.1 ~ 1.2 to reflect the additional expenses caused by uncertainty.

### 2.4 Determination of the objective function of dynamic adjustment of equipment maintenance cycle

The purpose of the dynamic adjustment of the equipment overhaul cycle is to minimize the cost of maintenance during the overhaul cycle of the equipment. The cost function consists mainly of the cost of repairing equipment failures $\sum_{I=1}^{n}U_{Ri}$ and planned maintenance direct costs $U_{m}$, so the optimization objective function is:


$$\begin{array}{c} minU=\sum_{I=1}^{n}U_{Ri}+U_{m} \end{array}$$ (3)

In the formula, minU denotes the dynamic adjustment objective function of the equipment overhaul cycle. The meaning of this objective function is to seek a maintenance cycle arrangement within the entire equipment operating time interval [0,T] that minimizes the sum of the planned maintenance cost $U_{m}$ and the fault maintenance cost $\sum_{I=1}^{n}U_{Ri}$.

### 2.5 Constraint setting

The constraints of the maintenance plan optimization model mainly include the coupling constraints between the total state of the maintenance plan, the maintenance start time and the daily state variables of the maintenance plan, the maintenance window period constraints, the daily maintenance quantity limitation constraints, the maintenance simultaneous constraints, the maintenance mutual exclusion constraints, and the above maintenance window period, the maintenance at the same time, the maintenance mutual exclusion, and the outage rules such as the area of the power protection, which are formulated by the grid dispatcher for the purpose of considering the safety of the grid and stable operation [14], so the maintenance plan optimization model proposed in this paper does not directly involve the grid security constraints.

(a) Coupling constraint between maintenance start time and general status of maintenance plan

$$\begin{array}{c} \varpi_{i}\leq x_{i}\leq \varpi_{i}T \end{array}$$ (4)

Where: $s_{i}$ is the start time of the maintenance program for the article i, as an integer variable. T is the total overhaul period, 1 year is 365d. Equation (4) indicates that when perform the overhaul program i, the range of values for the start time of the overhaul is [0,T]; If not performed, the service start time is 0; and $\varpi_{i}$ is the total status of the article overhaul program i.

(b) Coupling constraint between maintenance start time and daily status of maintenance plan

$$\begin{array}{c} f_{i,t}=\left\{ \begin{array}{c} @l@1s_{i}\leq t\leq s_{i}+T_{i}-1\\ 0t<s_{i}\|t>s_{i}+T_{i}-1 \end{array}\right. \end{array}$$ (5)

Where: $f_{i,t}$ is the status for the overhaul program i at the day t, and is 0−1 variable, 0 for no maintenance, 1 for maintenance. $T_{i}$ is the overhaul duration of the overhaul program i. Eq. (5) represents the maintenance state for maintenance program i during its maintenance interval $s_{i}\leq t\leq s_{i}+T_{i}-1$, the maintenance state outside the maintenance interval is 0. However, Eq. (5) is a nonlinear constraint, which needs to be linearized [15], and the specific transformation process is as follows.

By the inverse negation can get:


$$\begin{array}{c} \left\{ \begin{array}{c} @l@s_{i}\leq t\leq s_{i}+T_{i}-1\\ f_{i,t}=1 \end{array}\right.\Rightarrow \left\{ \begin{array}{c} @l@f_{i,t}=0\\ s_{i}\geq t+1\;or\;s_{i}\leq t-T_{i} \end{array}\right. \end{array}$$ (6)

Introducing the maintenance plan day state auxiliary 0–1 variable, the $z_{i,t}$, transforming equation (6) into:


$$\begin{array}{c} \left\{ \begin{array}{c} @l@s_{i}\geq t+1-(T+1)z_{i,t}-Ty_{i,t}-y_{i,t}\\ s_{i}\leq t-T_{i}+(T+T_{i})(1-z_{i,t})+y_{i,t}T+y_{i,t}T_{i} \end{array}\right. \end{array}$$ (7)

From Eq. (7): When $f_{i,t}=1$, equation (7) is constant and does not constitute a constraint, when $f_{i,t}=0$, $s_{i}\geq t+1\|t-T_{i}$ and one must hold, depending on the value of $z_{i,t}$, when $z_{i,t}=0$,$s_{i}\geq t+1$, when $z_{i,t}=1$, $s_{i}\leq t-T_{i}$.

The same leads to:


$$\begin{array}{c} \left\{ \begin{array}{c} @l@t<s_{i}\|t>s_{i}+T_{i}-1\\ f_{i,t}=0 \end{array}\right.\Rightarrow \left\{ \begin{array}{c} @l@f_{i,t}=1\\ s_{i}\leq t\leq s_{i}+T-1 \end{array}\right. \end{array}$$ (8)

Transforming equation (8) linearly into:


$$\begin{array}{c} Tf_{i,t}-T\leq t-s_{i}\leq T_{i}-1+(T-T_{i}+1)(1-f_{i,t}) \end{array}$$ (9)

From equation (9): when $f_{i,t}=0$, equation (9) is constant and does not constitute a constraint, when $f_{i,t}=1$, the $s_{i}\leq t\leq s_{i}+T-1$ established.

(2) Overhaul window period constraints

Service window means that the equipment can only be scheduled for service during its service window, subject to the following constraints:


$$\begin{array}{c} f_{i,t}=0,t\notin \Omega_{i,win} \end{array}$$ (10)

In the formula, $\Omega_{i,win}$ is the collection of maintenance windows for the maintenance program i.

(3) Daily overhaul quantity limit constraints

Considering the grid security and grid carrying capacity [16], it is necessary to limit the number of daily overhaul equipment to ensure the normal and stable operation of the grid, and it is necessary to set the daily overhaul number limitation constraint. Before setting the daily maintenance number constraint, the current maintenance number $n_{i}$ needs to be determined. The number of equipment failures is and specific failure analysis form is closely related [17], this paper assumes that the number of failures obey the Weibull distribution, Weibull distribution is often used to describe the failure of electronic and mechanical products law. Weibull distribution is a two-parameter distribution, its probability density f(t) is:


$$\begin{array}{c} f\left(t\right)=(\frac{mt}{\eta^{\text{2}}}{)}^{m-1}exp\left\{-(\frac{t}{\eta }{)}^{m}\right\} \end{array}$$ (11)

Where, η is the scale parameter, m is a shape parameter, t∈[0,T].

When m=1, the Weibull distribution is exponential, and when m=2, Weibull distribution is a Rayleigh distribution. The failure rate ζ(t) of the equipment when the failure pattern conforms to the Weibull distribution can be expressed as follows:


$$\begin{array}{c} \zeta \left(t\right)=(\frac{mt}{\eta^{\text{2}}}{)}^{m-1} \end{array}$$ (12)

Based on the historical fault time data of key equipment such as transformers, high-voltage switches, and circuit breakers in substations over the past decade, the maximum likelihood estimation method is used to fit the scale and shape parameters of the Weibull distribution. Taking the 1 # main transformer as an example, using MLE iteration to obtain m = 2.0, 95% confidence interval: [1.72, 2.28], η = 850 days, 95% confidence interval: [792, 908]. The p-value of the Kolmogorov Smirnov goodness of fit test is 0.23, which is greater than 0.05, indicating that the Weibull distribution can well describe the fault evolution law of the equipment. For equipment with limited fault data, the operating data of adjacent substations with the same voltage level and type of equipment is used as a reference, and proportional risk correction is made based on the service life of the equipment. The number of equipment failures $n_{i}$ during the i th prevention maintenance cycle is:


$$\begin{array}{c} n_{i}=\int_{\text{0}}^{t_{i}}\zeta_{i}\left(t\right)dt \end{array}$$ (13)

Among them, $\zeta_{i}\left(t\right)$ is the failure rate of equipment over a preventive maintenance cycle i, which is also the function of ageing factor $\tau_{i}$. By comparing and analyzing maintenance history data with changes in the failure rate, $\tau_{i}$ is estimated, and its recursive relationship reflects the degree of restoration of equipment status through different levels of maintenance. whose expression can be given by the following recurrence relation:


$$\begin{array}{c} \left\{ \begin{array}{c} @l@\lambda_{\text{1}}\left(t\right)=\lambda \left(t\right)\\ \zeta_{\text{2}}\left(t\right)=\zeta (t+t_{\text{1}}-\tau_{\text{1}}t_{\text{1}})\\ \vdots \\ \zeta_{i}\left(t\right)=\zeta (t+t_{i-1}-\tau_{i-1}t_{i-1})=\zeta t+\zeta \sum_{k=1}^{i-1}(t_{k}-t_{k}\tau_{k}) \end{array}\right. \end{array}$$ (14)

In the formula, k represents the total number of the ith equipment service cycles. The service life rollback factor in the above recursive relationship is used to quantify the extent to which different maintenance levels reduce the effective service life of equipment. Specifically, the retirement factor ∈ [0,1] represents the recovery ratio of the aging state of the equipment after the kth maintenance: a retirement factor of 0 represents the complete restoration of the equipment to its initial state after maintenance, and a retirement factor of 1 represents no improvement in aging caused by maintenance. Based on historical maintenance records and trends in failure rates, typical values are set for different maintenance levels: for L-level maintenance (such as visual inspection and simple parameter testing), it can only eliminate some potential defects, and the average effective service life of the equipment is reduced by about 15%, that is, the service life rollback factor is ≈ 0.85; For H-level maintenance (such as winding detection and replacement of key components), its recovery effect on equipment status is more significant, with an average reduction of about 40% in effective service life, that is, the service life rollback factor ≈ 0.60.

Based on the above recursive relationship, the formula for the number of failures $n_{i}$ can be rewritten as follows:


$$\begin{array}{c} n_{i}=\int_{\text{0}}^{t_{i}}\zeta_{i}\left(t\right)dt=\zeta t+\zeta \sum_{k=1}^{i-1}(t_{k}-\tau_{k}t_{k}) \end{array}$$ (15)

It is worth noting that the value of the shape parameter m in Equation (12) has a decisive impact on the pattern of fault evolution. Taking three typical cases,  m= 1.5, 2.0, and 2.5, as examples: when m=1.5, the equipment aging rate is relatively gradual, making it suitable to adopt a relatively longer maintenance interval; as m increases to 2.5, the equipment enters the wear-out phase more rapidly, and the failure rate curve becomes steeper, necessitating a corresponding shortening of the maintenance interval to control risks. This parameter sensitivity provides a theoretical basis for the differentiated setting of maintenance intervals—for similar equipment, if the m values obtained from fitting historical fault data differ, the preventive maintenance strategy should be adjusted accordingly. Similarly, changes in the scale parameter η affect the overall position of the failure rate curve; a smaller η indicates a shorter characteristic life, requiring more frequent maintenance interventions under the same conditions.

The Weibull distribution parameters are determined based on historical fault data from the equipment in this substation. Taking the transformer as an example, 27 fault records from three main transformers over the past 10 years were collected. Using the maximum likelihood estimation method, the shape parameter m=1.86 and the scale parameter η=1250 days were fitted. The K-S test yielded a p-value of 0.23 > 0.05, indicating that the Weibull distribution can adequately fit the actual fault data.

For equipment lacking sufficient historical data, parameters are estimated by referencing operational data from similar equipment in neighboring substations.

The age reduction factor $\tau_{i}$ is derived from an analysis of historical maintenance records: L-level maintenance typically reduces the equipment’s effective age by approximately 15% ($\tau_{i}\approx 0.15$), while H-level maintenance results in an average reduction of about 40% ($\tau_{i}\approx 0.40$).

Based on the current number of equipment failures $n_{i}$, set the constraints for setting the daily maintenance quantity limits as follows:


$$\begin{array}{c} \sum_{i=1}^{N}y_{i,t}\leq D_{t}-n_{i} \end{array}$$ (16)

In the formula, $D_{t}$ is the maximum number of daily overhauls for day t.

(4) Inspection while constrained

In order to avoid repeated outages, some maintenance equipment needs to meet the requirements of simultaneous maintenance [18], constrained by:


$$\begin{array}{c} \sum_{i}^{i\in \Omega_{i}}f_{i,t}\leq M \end{array}$$ (17)

In the formula, $\Omega_{i}$ is a collection of simultaneous overhaul programs. M is the number of maintenance plans in the set of simultaneous maintenance plans.

(5) Maintenance mutual exclusion constraints and repeated outage constraints

The simultaneous outage of certain maintenance equipment can lead to overloading of other equipment, and therefore the mutual exclusion constraint needs to be satisfied, i.e.,


$$\begin{array}{c} f_{i\in s_{\text{1}},t}+f_{i\in s_{\text{2}},t}\leq 1,s_{\text{1}},s_{\text{2}}\in \Omega_{j} \end{array}$$ (18)

In the formula, $\Omega_{j}$ is non-simultaneous overhaul of collections; overhaul of equipment $s_{\text{1}}$ and $s_{\text{2}}$ belonging to the overhaul mutually exclusive collection $\Omega_{j}$.

If the same equipment requires multiple overhauls [19], the corresponding maintenance plans must be executed separately and satisfy repeated outage constraints. These constraints are modeled in the same manner as maintenance mutual exclusion constraints, as follows:


$$\begin{array}{c} f_{i_{\text{1}},t_{a}}+f_{i_{\text{2}},t_{a}}\leq 1,i_{\text{1}},i_{\text{2}}\in \Omega_{a} \end{array}$$ (19)

Where, $\Omega_{a}$ is the maintenance plan set corresponding to equipment a;$f_{i_{\text{1}},t_{a}}$ is the maintenance plan $i_{\text{1}}$ of equipment a on day t, $f_{i_{\text{2}},t_{a}}$ is the maintenance plan a of equipment $i_{\text{2}}$ on day t.

(6) Overhaul timing constraints

Specific overhaul items are set up as prequel items with time intervals [20], for example, the maintenance start time of schedule j must be after the end time of schedule i, and the schedule j can only be executed after the maintenance end of schedule i meets a certain time interval:


$$\begin{array}{c} \left\{ \begin{array}{c} @l@s_{j}\geq s_{i}+\alpha_{i}T_{i}+\alpha_{i}W_{j}\\ s_{j}\leq T\alpha_{i} \end{array}\right. \end{array}$$ (20)

In the formula, $W_{j}$ is the interval between the end time of schedule i and the start time of schedule j.

(7) Reliability constraints

The objective function of this paper is to minimize the total maintenance cost of the system. However, in addition, the preventive maintenance cycle model based on a certain degree of reliability has a significant role to play in the smooth running of the production system. The general relationship between reliability and failure rate is as follows:


$$\begin{array}{c} E\left(t\right)=exp\left[-\int_{\text{0}}^{t}\lambda \left(t\right)dt\right] \end{array}$$ (21)

In the formula, E(t) indicates the reliability value.

From the above equation, the reliability expression for the operation of the equipment during each preventive maintenance cycle is given as:


$$\begin{array}{c} E_{i}\left(t_{i}\right)=exp\left[-\int_{\text{0}}^{t_{i}}\lambda_{i}\left(t\right)dt\right]=exp(-n_{i}) \end{array}$$ (22)

In the above equation, $n_{i}$ indicates the ith equipment the nth maintenance cycle.

After the nth preventive repair to the T running time, the reliability is expressed as follows:


$$\begin{array}{c} E(T-\sum_{i=1}^{n}t_{i}-\sum_{i=1}^{n}\theta_{i})=\underset{\text{0}}{\int^{T-\sum_{i=1}^{n}t_{i}-\sum_{i=1}^{n}\theta_{i}}}\lambda \left(t\right)dt \end{array}$$ (23)

In the above equation, $\lambda_{i}\left(t\right)$, $\theta_{i}$ both denote decision variables.

According to the above process, the dynamic adjustment target function contains several constraint variables, the optimization process of the dynamic adjustment of the equipment maintenance cycle is dynamically changing, and the optimization process of dynamic adjustment of the equipment maintenance cycle is very complex. In order to improve the operation timeliness, this paper uses the genetic algorithm and nonlinear planning to solve the method of dynamic adjustment objective function of the equipment maintenance cycle, to obtain the optimal equipment maintenance cycle dynamic adjustment parameters of the target function, to ensure that the planned maintenance cost and the failure cost are the lowest.

In terms of parameter settings, the genetic algorithm adopts a population size of 200 and 500 iterations, supplemented by an elitism strategy. Nonlinear programming employs the interior-point method for solution. Through multiple runs, this approach has been validated to exhibit stable convergence without premature convergence issues. Additionally, sensitivity tests were conducted on key parameters (such as failure rate parameters and cost coefficients) within a ± 20% range. The results indicate that the algorithm maintains good solution quality, with the optimized results fluctuating within ±3%, verifying the method’s strong robustness and engineering applicability.

## 3 Experimental analysis

Taking a 110kV substation as the experimental object, the 110kV substation is a kind of integrated automation substation, which is mainly responsible for normal power supply to the production plant, family living quarters, integrated companies, etc. The substation was completed and put into operation in October 2002. With the gradual expansion of production scale, it has experienced expansion in 2010 and technical transformation in 2014. The voltage level of the substation is 110KV/35KV/10KV. Under normal working conditions, Liantong Line I and Liantong Line II operate in parallel with 1 # and 2 # main transformers and the whole plant load through 1100 buscouple switches. The substation has one 35KV distribution room, two 10KV distribution rooms, five capacitors, and a total capacity of 13600KVAR, which ensures that the power factor meets the standard. In addition, there are three 50000KVA on load voltage regulating transformers (1 # and 2 # are in long-term operation, and 3 # are cold standby) with a total capacity of 150000 KVA, meeting the production and living power demand in the surrounding areas. This method is used to dynamically adjust the equipment maintenance cycle during the state operation and maintenance of the substation, and verify the practical application effect of this method. All key parameters (maintenance costs, failure cost, Weibull parameters, age reduction factors, algorithm settings, and priority weights) are derived from the substation’s historical operation and maintenance data over the past 3–10 years, calibrated via statistical fitting, pre-experiments, and expert experience.

To ensure the reproducibility of the algorithm, this section presents the key implementation details of the hybrid solution strategy.

(1) Decision variable encoding: The decision variables for each device include the start time of the maintenance (an integer ranging from 1 to 365, with 0 indicating no maintenance) and the maintenance level (binary, 0 for L-level and 1 for H-level). A mixed encoding method is used, with each device corresponding to a gene segment, and the total length of the chromosome being twice the number of devices. In this experiment, with 12 devices, the chromosome length is 24.
(2) Fitness function design: Based on the reciprocal of the objective function (total maintenance cost), a penalty term for violating constraints is added. For solutions that violate maintenance windows, daily maintenance quantity limits, mutual exclusivity, simultaneous maintenance, sequence, etc., a penalty coefficient is applied based on the degree of deviation, allowing infeasible solutions to be gradually eliminated during evolution. The larger the fitness value, the better the solution.
(3) Genetic algorithm parameters and convergence criteria: Population size 200, maximum number of iterations 500. Tournament selection, two-point crossover (probability 0.85), uniform mutation (probability 0.05) are used, and the two best individuals of each generation are retained. Convergence criterion: Terminate after 500 generations, or prematurely terminate after 50 consecutive generations of optimal fitness improvement less than 1e-4.
(4) Local optimization and convergence criteria for nonlinear programming: Using the optimal solution obtained by the genetic algorithm as the initial point, the interior point method is used for local optimization after continuous relaxation of the start time of maintenance. Convergence condition: Satisfy the KKT conditions (gradient tolerance 1e-6), or the relative change of the objective function in adjacent iterations is less than 1e-6, or reach the maximum iteration number 100 times. Finally, the rounded start time of maintenance is output.

### 3.1 Verification of equipment state perception capability

Substation state operation and maintenance process of equipment state perception is to realize the basis of the dynamic adjustment of equipment maintenance cycle, the substation state operation and maintenance, the substation high-voltage switchgear as an experimental object, the use of this paper’s method of sensing the substation operation and maintenance of the state of the state of the equipment, the results of the test are shown in Table 1.

**Table 1:** Equipment State Perception Results during Substation State Operation and Maintenance Process.

time	Current sensing/A	switch status
13:00	488.5	open
13:15	502	open
13:30	–	close
13:45	506.5	open
14:00	511.5	open
14:15	496.5	open
14:30	424.7	open
14:45	–	close
15:00	–	close
15:15	318.5	open
15:30	508.1	open

Note: “-” in the above table represents “none.”

According to the analysis results in Table 1, we can see that the method in this paper can accurately obtain the HV switch current status value and current switch status during the operation and maintenance of substation status at different times. These data can not only monitor the operation status of equipment in real time, but also provide accurate operation and maintenance status for subsequent dynamic adjustment of equipment maintenance cycle. Further, from these data, we can learn about the real-time operation status of the equipment, whether there are exceptions, and the problems that may occur. Through this information, we can take corresponding measures in time to avoid greater impact of equipment failure on the entire power system. In addition, it is also proved from the side that the method in this paper has a strong ability to dynamically adjust the maintenance cycle of substation equipment. According to the actual operation state of the equipment, the maintenance cycle of the equipment can be flexibly adjusted to ensure that the equipment operates in the best state and improve the operation efficiency and safety of the equipment. In conclusion, the method in this paper has significant advantages and strong ability in the dynamic regulation of substation equipment maintenance cycle. The intelligent operation and maintenance of the equipment can be realized by obtaining the operation status data of the equipment in real time, and the operation efficiency and security of the equipment can be improved.

### 3.2 Analysis of dynamic adjustment results and priority rules for equipment maintenance cycle

The substation 12 equipment as an experimental object, the use of this paper’s method of the 12 substation equipment maintenance cycle for dynamic adjustment, adjustment results shown in Table 2.

**Table 2:** Dynamic Adjustment Test Results of Equipment Maintenance Cycles in 12 Substations.

Substation equipment	Maintenance cycle time	Dynamic adjustment time
High voltage switch	04/06-04/07	04/06
transformer	04/06-04/09	04/07-04/08
Circuit breaker	04/07-04/09	04/08
Overcurrent Protection Device	04/08-04/10	04/09-04/10
Overvoltage protection device	04/06	04/05
capacitor	04/11-04/12	04/09-04/11
reactor	04/12-04/14	04/12-04/13
current transformer	04/13-04/16	04/13-04/15
voltage transformer	04/13-04/16	04/14-04/16
arc-suppression device	04/15	04/17
Grounding device	04/17	04/17
disconnector	04/18-04/19	04/18-04/19

As can be seen from Table 2, the 12 devices in this substation exhibited significant time conflicts in the original maintenance schedule. Taking the high-voltage switch and the transformer as examples: the original maintenance period for the high-voltage switch was 04/06–04/07, and for the transformer it was 04/06–04/09, resulting in a complete overlap during the 04/06–04/07 period. Such conflicts lead to strain on operation and maintenance resources, and the simultaneous outage of critical equipment increases the operational risk to the power grid. After dynamic adjustment using the method proposed in this paper, the maintenance periods for all devices were properly arranged, completely eliminating the time conflicts.

The core mechanism by which the proposed method resolves time conflicts is a multi-dimensional priority comprehensive evaluation system. During the objective function optimization process, the algorithm incorporates clear priority determination rules, mainly comprising the following levels:

(1) Equipment Criticality Priority

The importance of equipment within the power system is the primary consideration. Based on the scope and severity of the impact of equipment failure on the safe and stable operation of the power grid, this paper defines equipment criticality levels:

Level 1 Critical Equipment: Transformers, busbars, etc. Failures lead to large-scale blackouts, require long recovery times, and have the greatest impact on the system, thus receiving the highest priority.

Level 2 Critical Equipment: Circuit breakers, high-voltage switches, etc. Failures have a relatively wide impact range, but load can be partially transferred through backup measures, thus receiving the second-highest priority.

Level 3 General Equipment: Capacitors, reactors, instrument transformers, etc. Failures have a relatively local impact and thus receive lower priority.

In the case study of Table 2, the transformer, as the core equipment of the substation, has a far greater impact on the system than the high-voltage switch. The algorithm schedules the transformer for maintenance on 04/07–04/08 (occupying 2 days) and the high-voltage switch separately on 04/06 (occupying 1 day). This decision is based precisely on the higher criticality priority of the transformer—by staggering the times, it ensures that both critical devices are not out of service simultaneously, reducing system operational risk. This arrangement embodies the core principle of “prioritizing critical equipment.”

(2) Equipment Health Status Priority

Based on real-time condition monitoring data, the algorithm evaluates the current health status of the equipment. A health score (0–100) is used to quantify the equipment’s condition, where a lower score indicates a worse health state and higher maintenance urgency. During optimization, equipment with a health score below a threshold (e.g., 80) receives higher maintenance priority.

Taking the transformer and high-voltage switch as examples: condition monitoring data shows abnormal oil chromatography for the transformer (health score 78), while all indicators for the high-voltage switch are normal (health score 85). Therefore, even without considering the criticality difference, the transformer should be prioritized for maintenance. After integrating the criticality weight (0.4) and the health status weight (0.3), the algorithm determines that the transformer has a higher priority than the high-voltage switch, ultimately scheduling it in a more time slot (2 days) for thorough maintenance.

(3) Maintenance Window Urgency Priority

The available time window for scheduling equipment maintenance is constrained by factors such as power grid operation modes, load characteristics, and power supply reliability requirements. Equipment whose window is about to close receives higher priority. For example, the original maintenance window for the overvoltage protection device was only 04/06. Missing this window would mean waiting for the next window cycle (possibly several months later). The algorithm advances its maintenance to 04/05, scheduling it separately. This avoids conflicts with devices like the high-voltage switch and transformer while ensuring the maintenance task is completed within its window.

(4) Maintenance Duration and Resource Occupancy Priority

Equipment requiring longer maintenance durations needs to start earlier to avoid occupying subsequent maintenance resources and to prevent delays that could lead to conflicts with other devices. For instance, the original maintenance period for the capacitor was 04/11–04/12 (2 days). After adjustment, it was moved earlier to 04/09–04/11 (3 days). This adjustment is based on two considerations: first, health monitoring of the capacitor shows increased dielectric loss, requiring more maintenance time; second, by scheduling it earlier, it avoids the peak maintenance period for multiple devices like the reactor and instrument transformers from 04/12–04/14, achieving resource balancing.

(5) Mutual Exclusion and Synergy Constraints

For equipment that must be maintained simultaneously (e.g., associated devices on the same busbar, equipment requiring coordinated outages), the algorithm forces scheduling within the same time period. For mutually exclusive equipment (e.g., devices whose simultaneous outage would cause overload or reduce power supply reliability), the algorithm forces staggering. In Table 2, the current transformer (04/13–04/15) and the voltage transformer (04/14–04/16) have partial overlap, which meets the requirement for synergistic maintenance—they typically require coordinated outages but not necessarily perfect simultaneity. Appropriate overlap satisfies the synergy requirement while avoiding excessive resource concentration.

The five categories of priority rules mentioned above are not used in isolation but are integrated into the objective function through a weighted multi-attribute decision-making method. The specific decision process is as follows:

(1) Hard Constraint Filtering: First, ensure all candidate solutions satisfy mandatory conditions such as maintenance windows, mutual exclusion constraints, simultaneous constraints, sequence constraints, and daily maintenance quantity limits.
(2) Multi-Index Weighted Scoring: For feasible solutions, calculate the comprehensive priority score: $P=\omega_{k}\cdot K+\omega_{h}\cdot H+\omega_{w}\cdot W+\omega_{d}\cdot D$

Where K is the equipment criticality score (Level 1 critical equipment = 100, Level 2 = 80, Level 3 = 60); H is the health status score (100 – health score); W is the window urgency score (the fewer days remaining until the window closes, the higher the score); D is the duration score (the longer the duration, the higher the score). The weight coefficients $\omega_{k}$, $\omega_{h}$, $\omega_{w}$, $\omega_{d}$ are determined through the Analytic Hierarchy Process combined with expert experience. In this paper, the values are $\omega_{k}=0.4$, $\omega_{h}=0.3$, $\omega_{w}=0.2$, $\omega_{d}=0.1$.

(3) Optimization Solution: Genetic algorithms and nonlinear programming cooperate in the search. Under the premise of satisfying all constraints, maintenance tasks with higher comprehensive priority scores are scheduled preferentially, while simultaneously minimizing the total maintenance cost.

Taking the resolution of the conflict between the transformer and the high-voltage switch as an example, the comprehensive priority scores for both are calculated:

- Transformer: Criticality score 100 (Level 1), Health status score 22 (100−78), Window urgency score 60 (10 days remaining in window), Duration score 80 (2-day duration) → P=0.4×100+0.3×22+0.2×60+0.1×80=66.6
- High-voltage switch: Criticality score 80 (Level 2), Health status score 15 (100−85), Window urgency score 60 (10 days remaining in window), Duration score 40 (1-day duration) → P=0.4×80+0.3×15+0.2×60+0.1×40=52.5

The comprehensive priority score of the transformer (66.6) is significantly higher than that of the high-voltage switch (52.5). Therefore, the algorithm prioritizes the maintenance needs of the transformer, scheduling it in a more time slot (04/07–04/08), while the high-voltage switch is scheduled separately on 04/06. This decision aligns with the principle of “prioritizing critical equipment” and also reflects the core idea of condition-based maintenance, which is “dynamic adjustment based on health status.”

Through the comprehensive application of the above priority rules, the proposed method can effectively resolve time conflicts in complex multi-equipment maintenance scheduling and achieve optimal allocation of maintenance resources. The results in Table 2 show that the adjusted schedule for the 12 devices not only has no time conflicts but also features reasonably shortened maintenance periods for high-priority critical equipment such as the high-voltage switch, transformer, and circuit breaker, fully demonstrating the core role of the priority rules in conflict resolution.

### 3.3 Verification of equipment reliability improvement effect

**Fig 3:**
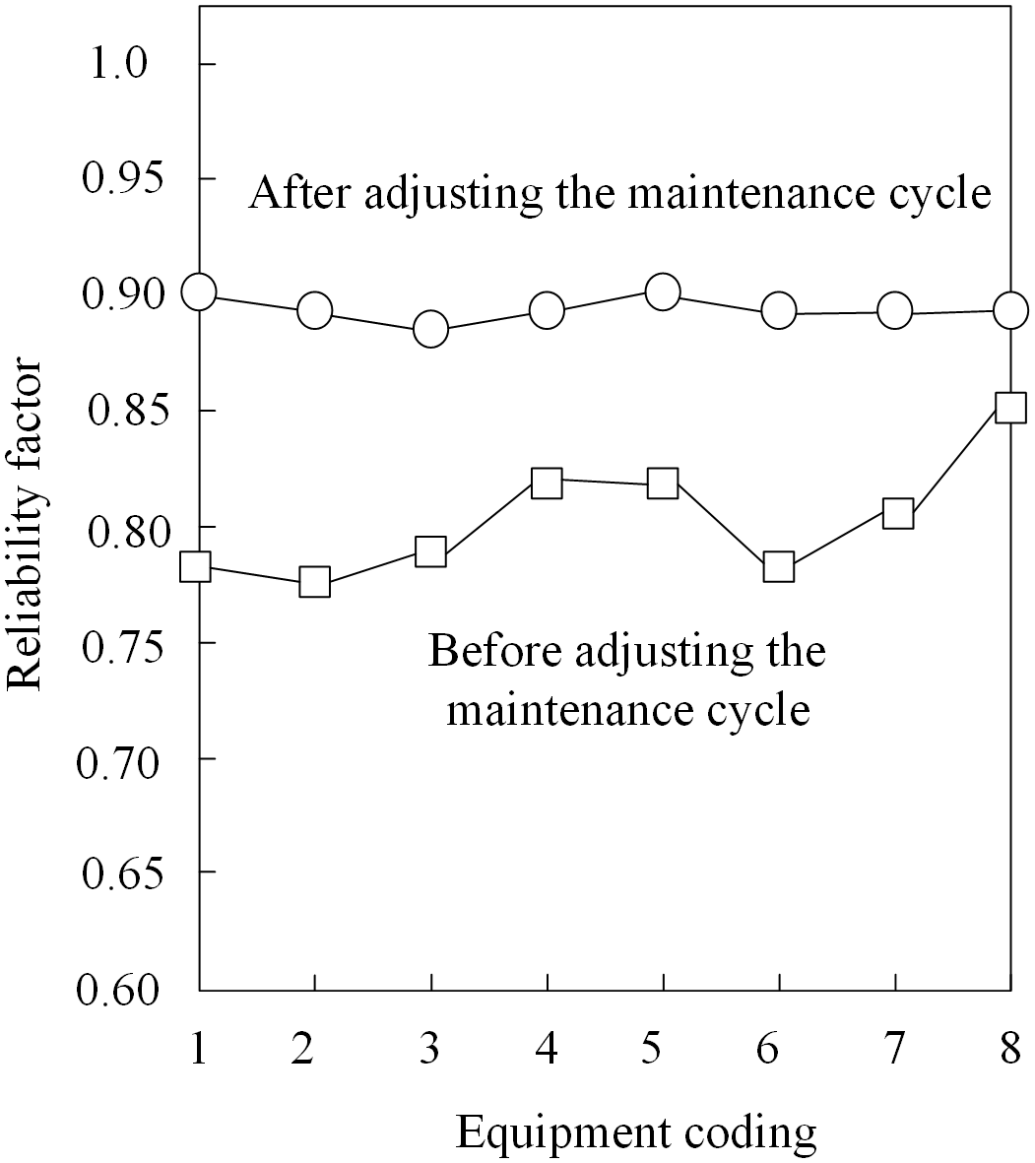
Reliability coefficient of substation equipment.

With 8 substation equipment as the experimental object, using this paper’s method of dynamic adjustment of its maintenance cycle, the reliability coefficient of the equipment as a measure of indicators, to verify the practical application of this paper’s method. The test results are shown in Fig 3.

Based on the analysis results in Fig 3, the reliability coefficients of the eight substation devices have significantly improved after adjusting their maintenance cycles using the proposed method. After adjustment, the reliability coefficients of all eight devices remained around 0.9, with an average value of 0.92, representing an improvement of approximately 10.8% compared to the pre-adjustment average (approximately 0.83). This result indicates that dynamic adjustment of maintenance cycles enables devices to operate in a healthier state, effectively reducing the risk of failures.

The improvement in equipment reliability can be attributed to two main factors: first, the reasonable shortening of maintenance cycles ensures more timely maintenance, preventing the accumulation of potential faults; second, the health-based priority rules ensure that devices in poorer health conditions receive maintenance resources first, achieving precise maintenance. Taking Device 3 as an example, its reliability coefficient was relatively low before adjustment (approximately 0.78). By scheduling maintenance earlier and appropriately extending the maintenance duration, its reliability improved to 0.91, demonstrating a particularly significant enhancement.

**Fig 4:**
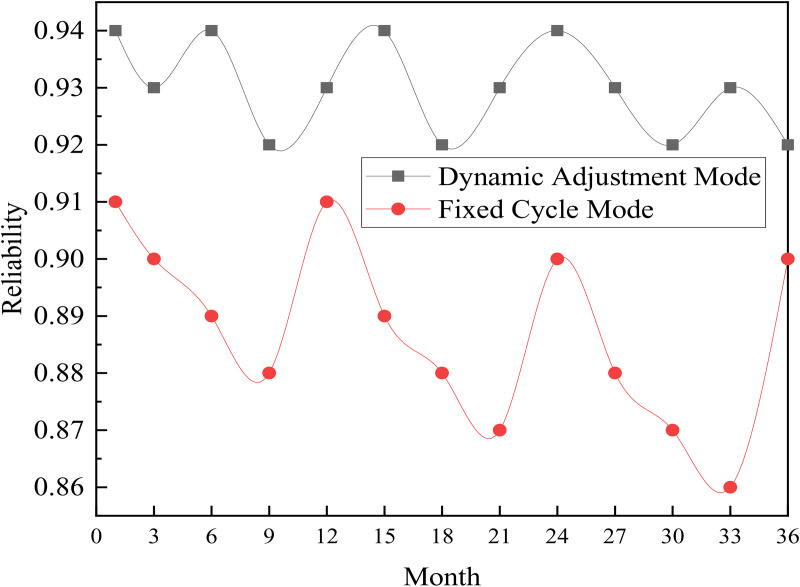
Comparison of Long-Term Reliability Curves for Transformers.

To further validate the long-term advantages of the proposed method, a transformer was selected as a typical device to compare and analyze the trends in reliability under two modes: dynamic adjustment of maintenance cycles and fixed-cycle maintenance. The results are shown in Fig 4. The fixed-cycle mode adopts the traditional strategy of annual periodic maintenance, while the dynamic adjustment mode employs the proposed method to dynamically adjust maintenance cycles based on the real-time condition of the equipment.

As shown in Fig 4, over the 36-month observation period, the reliability curve of the transformer under the dynamic adjustment mode consistently remains above that of the fixed-cycle mode. In the fixed-cycle mode, the equipment’s reliability exhibits a distinct “sawtooth” fluctuation pattern over time: reliability recovers after each maintenance, but due to the fixed maintenance schedule, it fails to promptly respond to the gradual deterioration of the equipment’s condition. This results in significant reliability declines between maintenance intervals, with the lowest point dropping to approximately 0.85. In contrast, under the dynamic adjustment mode, the algorithm schedules maintenance appropriately based on the real-time condition of the equipment, enabling timely intervention before reliability falls below the threshold. This ensures that reliability is consistently maintained at a high level above 0.90.

Further analysis of the mean reliability and standard deviation under the two modes reveals the following: under the dynamic adjustment mode, the average reliability of the transformer is 0.93, with a standard deviation of 0.018; under the fixed-cycle mode, the average reliability is 0.88, with a standard deviation of 0.035. The dynamic adjustment mode not only achieves a higher average reliability but also exhibits smaller fluctuations, indicating that this method can more stably maintain the equipment’s health status and effectively avoid significant reliability fluctuations caused by improper maintenance timing.

Moreover, from a long-term perspective, the equipment reliability under the fixed-cycle mode shows a gradual declining trend (from 0.91 to 0.86 over 36 months), while under the dynamic adjustment mode, reliability remains relatively stable within the range of 0.92–0.94, with no significant decline observed. This result demonstrates that the dynamic adjustment method, by accurately determining the optimal timing for maintenance, can effectively slow down the equipment aging process and achieve comprehensive health management throughout the equipment’s lifecycle.

The analysis of the long-term reliability curves above fully illustrates that, compared to the traditional fixed-cycle maintenance mode, the dynamic adjustment method proposed in this paper not only enhances equipment reliability after a single maintenance but also consistently maintains high reliability levels across multiple maintenance cycles, demonstrating significant and enduring advantages.

### 3.4 Analysis of maintenance frequency changes

To the annual maintenance number of equipment as an experimental object, using the method of this paper on the substation 10 equipment maintenance cycle adjustment, statistics of the 10 substations in the 12-month maintenance number of changes, test results shown in Table 3.

**Table 3:** Maintenance frequency of substation equipment within 12 months (times).

Substation equipment coding	Before adjusting the maintenance cycle	After adjusting the maintenance cycle
1	13	15
2	9	12
3	8	15
4	15	18
5	21	25
6	22	25
7	19	25
8	17	20
9	20	25
10	18	20

Based on the analysis results in Table 3, after adjusting the maintenance cycle using the method proposed in this paper, the average number of maintenance actions for the 10 pieces of equipment over 12 months increased from 16.2 to 20.5, representing an increase of 26.5%. This change reflects a reasonable shortening of the maintenance cycle and an improvement in the timeliness of operational responses.

There are differences in the increase in maintenance frequency among different pieces of equipment: Equipment 3 showed the largest increase, from 8 to 15 maintenance actions (an increase of 87.5%). This is consistent with its relatively low reliability before adjustment (approximately 0.78 for Equipment 3 in Fig 3), indicating that the algorithm prioritized equipment in poorer health by increasing their maintenance frequency. In contrast, Equipment 6 showed a smaller increase, from 22 to 25 maintenance actions (an increase of 13.6%), as it already maintained a higher maintenance frequency before adjustment. This differentiated adjustment embodies the core concept of condition-based maintenance—the allocation of maintenance resources should align with the actual needs of the equipment, rather than adhering to a rigid “one-size-fits-all” fixed cycle.

### 3.5 Comparative analysis of economic costs

The economic cost of substation equipment maintenance as a measure of indicators, transformers, capacitors, reactors, arc extinguishing devices, as an experimental object, use this paper’s method of dynamic adjustment of its maintenance cycle, in order to make the experimental results more adequate, while using the literature [4] of the state-based maintenance of equipment life cycle adjustment method, literature [5] of the hydroelectric power plant equipment condition monitoring and maintenance cycle adjustment method, literature [6] of the equipment maintenance scheduling static adjustment method, literature [7] of the deep reinforcement learning-based equipment dynamic maintenance cycle adjustment model and literature [8] of the cloud computing-based equipment maintenance scheduling static adjustment method, the experimental results are shown in Fig 4.

To ensure the fairness of the comparison, the implementation details and parameter settings of each baseline method are as follows:

(1) Method from Reference [4]: Reproduced according to the original description. Equipment health status is divided into four levels: normal, attention, abnormal, and severe, corresponding to maintenance cycle adjustment coefficients of 1.0, 0.8, 0.6, and 0.4, respectively. Health status assessment adopts the health scoring system from Section 3.2 of this paper, with thresholds set at 90 points (normal), 80 points (attention), 70 points (abnormal), and 60 points (severe). Other parameters (e.g., cost parameters, failure rate parameters) are consistent with the method proposed in this paper.
(2) Method from Reference [5]: Driven by real-time condition monitoring data, maintenance is triggered when equipment condition parameters exceed warning thresholds. In this paper, voltage deviation exceeding ±5%, current fluctuation exceeding ±10%, and partial discharge exceeding 100 pC are set as trigger conditions. The maintenance cycle is shortened to 0.5 times the original plan. Threshold settings are adjusted based on the original reference and actual operational data from the substation.
(3) Method from Reference [6]: Static priority is assigned based on equipment urgency, determined by equipment criticality and operating time. In this paper, equipment criticality levels are defined as in Section 3.2 (Level 1 critical equipment: priority 3, Level 2: priority 2, Level 3: priority 1). Operating time is measured in years. The comprehensive priority = criticality level × operating time. Maintenance is scheduled in descending order of priority without dynamic optimization.
(4) Method from Reference [7]: The Deep Deterministic Policy Gradient algorithm is employed. The neural network structure consists of three fully connected layers (128 neurons per layer), with ReLU activation function, Adam optimizer, learning rate 0.001, discount factor 0.9, experience replay pool capacity 10,000, batch size 64, and training episodes 2,000. The state space includes equipment health score, operating time, and historical failure count. The action space is the maintenance cycle adjustment coefficient (continuous value in the range 0.5–2.0). The reward function is the negative total maintenance cost.
(5) Method from Reference [8]: A cloud computing architecture is adopted for centralized optimization. The optimization algorithm is Particle Swarm Optimization, with population size 100, iteration count 300, inertia weight 0.7, and learning factors c1 = c2 = 1.5. The objective function is the same as in this paper, but mutual exclusion, sequencing, and other complex constraints among multiple equipment are not considered.

All comparative experiments are conducted under the same hardware environment (Intel Core i7-10750H CPU @2.60GHz, 16GB RAM) and with identical cost parameters and failure rate parameters to ensure comparability of the results.

**Fig 5:**
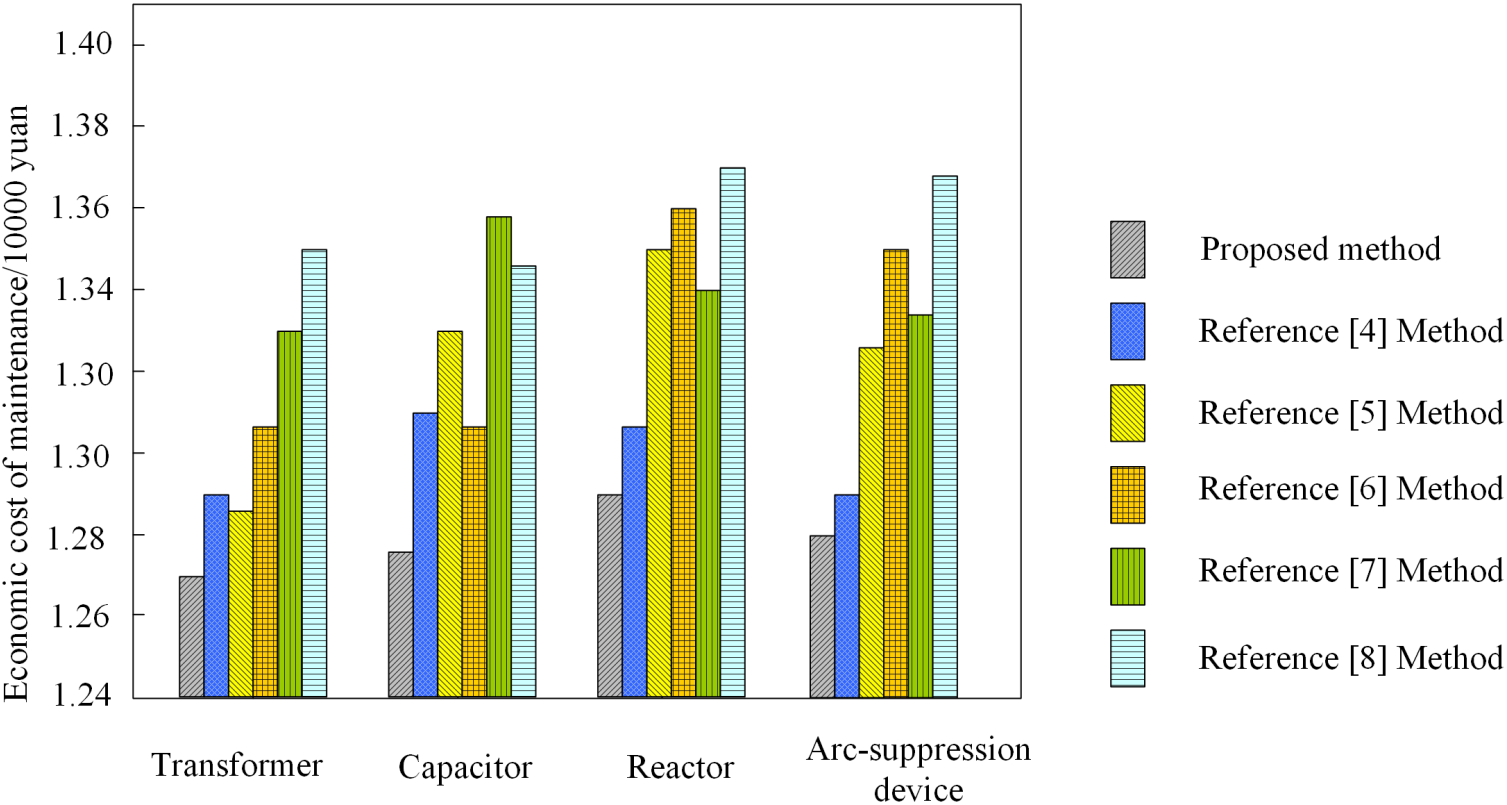
Economic cost of equipment maintenance in different substations.

According to Fig 5, after adjusting the maintenance cycle of substation equipment, the economic cost of maintenance of different substation equipment is the lowest. This shows that this method can effectively reduce the economic cost of substation equipment maintenance and improve the economy of equipment. In addition, the application effect of literature [4] method is also good, and the economic cost of maintenance of different substation equipment is also low. In contrast, the application effect of the method in literature [8] is the worst, and the economic cost of equipment maintenance in different substations is more than 13600 yuan at most. This shows that the method in literature [8] has no significant effect on reducing the economic cost of substation equipment maintenance. In summary, the method in this paper has the best application effect among the six methods, which can effectively reduce the economic cost of substation equipment maintenance, and the application effect is more significant.

The economic advantages of the method proposed in this paper stem from two key aspects: first, through precise condition monitoring and priority-based rules, maintenance resources are allocated to the equipment and timing where they are most needed, thereby avoiding ineffective or excessive maintenance; second, by leveraging the synergistic optimization of genetic algorithms and nonlinear programming, the total cost is minimized while satisfying reliability constraints. In comparison, the method in reference [8] incurs the highest cost, indicating that purely cloud-based computational approaches are less efficient in handling complex constraint optimization problems. The method in reference [4] demonstrates better performance, but its state-based adjustment mechanism fails to adequately account for conflicts and resource constraints among multiple devices, leaving room for further optimization.

### 3.6 Analysis of maintenance schedule balancing

**Fig 6:**
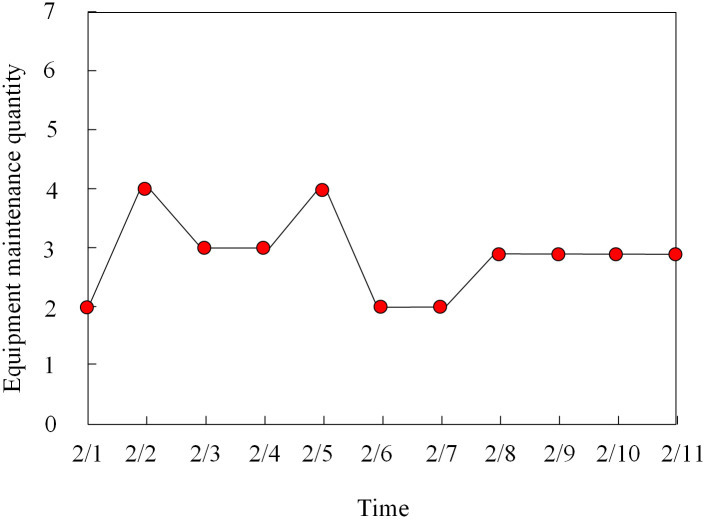
Balance of maintenance cycle adjustment.

To further verify the practical application effect of the method in this paper, the balance of the maintenance cycle regulation of substation equipment is used as a measurement index to analyze the number of maintenance equipment in different time periods, and the results are shown in Fig 6.

Analysis of Fig 6 shows that, using the method of this paper on the substation equipment maintenance cycle adjustment, the substation daily maintenance of the number of equipment between 2–4, the results show that the substation on the number of equipment maintenance arrangements are more reasonable, will not cause too much maintenance equipment overhaul task conflict phenomenon, will not cause too little maintenance equipment, maintenance personnel idle. The above results show that after the application of this method, the balance of substation equipment maintenance is better, and the method of this paper has a stronger ability to regulate the maintenance cycle of substation equipment.

### 3.7 Sensitivity analysis of cost parameters

To validate the rationality of the cost model parameters, the transformer is selected as the analysis object. The key cost parameters are varied within a range of ±20%, and the resulting changes in the optimization outcomes are observed, as presented in Table 4.

**Table 4:** Results of Cost Parameter Sensitivity Analysis.

Parametric variation	Annual average total cost variation	Cycle of maintenance
$U_{mL}$±20%	±3.0%	No change
$U_{mH}$±20%	±5.2%	± 2–3 days
$\sum_{I=1}^{n}U_{Ri}$±20%	±7.1%	±4–5 days

The results indicate that when the cost parameters fluctuate within a ± 20% range, the variation in the average annual total cost remains within ±8%, and the change in the maintenance cycle is confined to within ±5 days. This demonstrates that the algorithm exhibits good robustness to parameter variations, thereby verifying the reasonableness of the cost model parameter settings.

## 4 Conclusion

This article proposes a dynamic adjustment algorithm for the maintenance cycle of intelligent substation equipment. This algorithm constructs an integrated optimization framework that integrates multi-source state perception, fine cost modeling, and multidimensional constraints, and designs a two-stage solution strategy of collaborative genetic algorithm and nonlinear programming to achieve dynamic and precise adjustment of maintenance cycles driven by real-time operating status. The experimental results show that after adjusting the maintenance cycle of the equipment using the proposed method, the reliability coefficient of the equipment remains stable at around 0.9, with an average value of 0.92. In the dynamic adjustment mode, the average reliability of the transformer is 0.93, with a standard deviation of 0.018; In the fixed cycle mode, the average reliability of the transformer is 0.88, with a standard deviation of 0.035. After adjusting the maintenance cycle using our proposed method, the average maintenance frequency increased from 16.2 times to 20.5 times, with a growth rate of 26.5%. When the cost parameter fluctuates within the range of ± 20%, the change in the annual average total cost is controlled within ± 8%, and the change in maintenance cycle is limited to ± 5 days.

This study identifies the following directions for further exploration: First, incorporating environmental factors such as temperature and humidity into the dynamic adjustment model for maintenance cycles, investigating the correlation mechanisms between environmental factors and equipment aging processes, and further enhancing the algorithm’s adaptability in complex environments. Second, conducting application validation on larger-scale substation systems. While the current study validates the algorithm’s effectiveness based on experiments with 12 pieces of equipment in a 110kV substation, subsequent work will involve actual substation data with more equipment to further test the algorithm’s industrial applicability and optimize the convergence efficiency of genetic algorithms for large-scale problems.

In summary, the algorithm proposed in this paper holds certain theoretical value and application potential in the field of substation condition-based maintenance. Future research will focus on the aforementioned directions to advance the technology toward engineering and systematic development.
